# Attitudes and influencing factors associated with smoking cessation: An online cross-sectional survey in China

**DOI:** 10.18332/tid/166108

**Published:** 2023-06-26

**Authors:** Jian-Hua Wang, Yu-Feng Yang, Shi-Lei Zhao, Hai-Tao Liu, Lei Xiao, Li Sun, Xi Wu, Dong-Chao Yuan, Li-Yao Ma, Bao-Zhao Ju, Jian-Ping Liu

**Affiliations:** 1 Institute of Chinese Medicine, Liaoning University of Traditional Chinese Medicine, Shenyang, China; 2 Department of Anesthesia, General Hospital of Northern Theater Command, Shenyang, China; 3 Department of Cardiovascular Medicine, The People's Hospital of Liaoning Province, Shenyang, China; 4 Affiliated Hospital, Liaoning University of Traditional Chinese Medicine, Shenyang, China; 5 College of Acupuncture, Moxibustion, Liaoning University of Traditional Chinese Medicine, Shenyang, China; 6 School of Pharmaceutical Sciences, Liaoning University of Traditional Chinese Medicine, Dalian, China; 7 Center for Evidence-Based Chinese Medicine, Beijing University of Chinese Medicine, Beijing, China

**Keywords:** smoking cessation, attitudes, influencing factors, online cross-sectional survey

## Abstract

**INTRODUCTION:**

Quitting smoking, the critical path to reach the global targets of reducing tobacco use, can bring major and immediate health benefits to smokers. Exploring factors that help individuals to quit smoking is of great importance. The present study explored influencing factors on smoking cessation, in order to provide comprehensive reference for tobacco control policies.

**METHODS:**

Ex-smokers and current smokers were recruited online in this cross-sectional survey, from 1 October to 31 November 2022, in China. The observational data were collected using a questionnaire to collect information with respect to sociodemographic characteristics of smokers, attitudes towards smoking cessation, details of smoking cessation, and different potential factors related to smoking cessation through open-ended questions.

**RESULTS:**

A total of 638 smokers from 30 provinces were recruited as eligible respondents, with a mean age of 37.3 ± 11.7 years and a mean smoking history of 15.9 ± 13.7 years. The percentage of males was 92.3%. Of the 638 respondents, only 3.9% had no intention to stop smoking. Among 155 subjects who had quitted smoking successfully, willpower (55.5%) was considered as the most important contributing factor. Among 365 subjects who tried to quit but failed, lack of willpower (28.2%), tobacco dependence (16.2%), influence of surrounding smokers or smoking environments (15.9%), bad moods (9.9%), stress from work or life (7.9%), habits (7.1%), socialization (4.1%), and easy availability of tobacco (2.7%) were considered as the adverse factors leading to failure in quitting smoking.

**CONCLUSIONS:**

Willpower and support from family members were the vital factors that lead to successful smoking cessation. Future tobacco control policies should also focus on addressing withdrawal symptoms and creating smoke-free environments as well as other factors.

## INTRODUCTION

Tobacco smoking remains a worldwide public health issue, and it is the leading cause of avoidable premature mortality and diseases^1^. An estimated 1.3 billion people worldwide use tobacco which kills >8 million people every year^2^. China is one of the main countries for tobacco production and consumption^3^. The number of smokers in China exceeds 300 million and the smoking rate among people aged ≥15 years was 26.6% in 2018^4^. One prospective cohort study has predicted that the annual number of deaths caused by smoking in China would rise from 1 million in 2010 to 3 million in 2050 without active intervention^5^.

Quitting smoking, the critical path to reach the global targets of reducing tobacco use^6^, can bring major and immediate health benefits to smokers at any stage or age^7^. Under WHO’s Framework Convention on Tobacco Control (FCTC), China has developed Chinese Clinical Smoking Cessation Guidelines to help quit smoking^8^. In addition to this, there are a wide variety of evidence-based smoking cessation therapies such as nicotine replacement therapy, cognitive-behavioral therapy, and psychological support. However, despite the development of cessation strategies and interventions, the cessation rate is still very low with a high risk of relapse^9^. Smoking is a complex behavior involving physical, psychological, social and other factors^10^, and the subjective initiative of smokers plays a prominent role in the process of quitting smoking^11^. So, analyzing factors reported by smokers that influence smoking cessation can assist in developing targeted intervention strategies. Previous studies have provided limited findings based on research hypothesis, certain factors for smoking cessation have been studied separately, including education, region, smoking-related knowledge, noticing label warnings and advertisements, exposure to other smokers or tobacco-related messages, diseases, tobacco tax, and psychological distress^12-14^. To our knowledge, no existing studies have studied potential influencing factors of smoking cessation through open-ended questions.

Therefore, the present study conducted a survey in the form of open-ended questions to collect data on all influencing factors of smoking cessation, allowing smokers to express their true feelings freely. The research results can provide more comprehensive reference for subsequent studies and be used to design a more comprehensive and targeted tobacco control policies focused on increasing success rates in quitting smoking.

## METHODS

### Study design and participants

This was a cross-sectional online survey carried out among smokers in China. Study subjects were recruited on the WeChat, a cloud-based social media networking application, from 1 October to 31 November 2022. Participants who were current or former smokers were eligible to participate in the study. The respondents with incomplete data in the questionnaire were excluded from the study. Smokers were defined as those who had smoked ≥100 cigarettes in their lifetime^15^. Quitting smoking successfully was defined as maintaining abstinence from smoking for at least six months. STROBE scale^16^ has been drawn in this study to improve the quality of reporting (Supplementary file). At the beginning of the study, participants were informed about the purpose of the study. And respondents participated in this survey completely voluntarily. All procedures performed in our survey were in accordance with the 1964 Helsinki Declaration. Any personally identifiable information was not documented.

### Questionnaire

A questionnaire was designed to collect relevant data. It contained 14 questions from 5 items: sociodemographic characteristics of smokers (such as gender, age, province of residence, tobacco use information), attitudes towards smoking cessation, characteristics of smoking cessation, factors that promote smoking cessation, and factors that hinder smoking cessation. Most questions were designed in open-ended format to avoid limiting the diversity of answers. Of these 14 questions, questions 1–6 needed to be answered by all subjects, questions 7–8 by subjects who had a quit attempt, questions 9–11 by subjects who had quitted smoking successfully, questions 12–14 by subjects who tried to quit but failed.

Anonymous online questionnaires were distributed to respondents and then collected. These questionnaires could be filled in and submitted online in 3 to 5 minutes.

### Sample size

Adopting a simple random sampling method, the sample size in this survey was calculated using the sample size estimation formula for the individual overall rate:

N=(μ_α_/Δ)^2^ × p (1-p)

where p=0.28^17^, Δ was set at 0.26/6, α=0.05 and μ_α_=1.96^18^. This gave a value of N=412, but in order to include a possible 20% failure rate of the questionnaire, the total sample size needed to be ≥495.

### Statistical analysis

Respondents with missing data were excluded and were not analyzed. Descriptive statistical analysis was conducted using SPSS (release 2019, Version 26.0, IBM, Armonk, NY, USA) and WPS (version 11.1.0.12598, WPS Office, China). Categorical variables are presented as frequencies and percentages, while continuous variables are given as means with standard deviation (SD). A baseline characteristics comparison of respondents who succeeded in quitting smoking and those who did not was carried out by using χ^2^ tests and t-tests.

## RESULTS

### Description of participants

**Figure 1:**
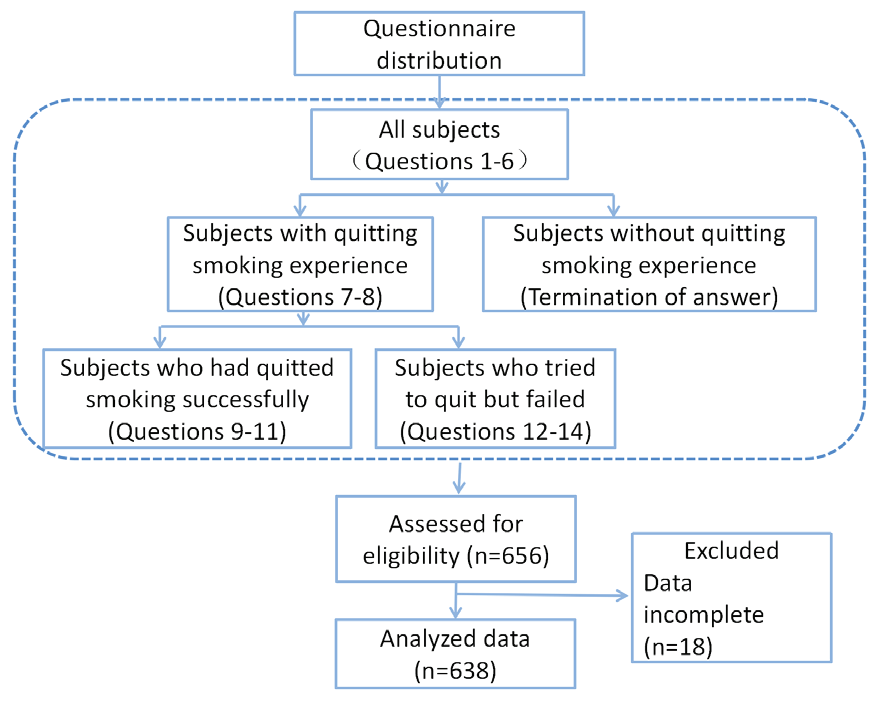
The flowchart of data collection related to attidutes and influencing factors associated with smoking cessation from 1 October to 31 November 2022 in China

Of the 656 respondents who submitted the questionnaires, 638 (97.3%) filled in the questionnaires completely. Of the 638 final participants, 520 (81.5%) had at least one previous quit attempt which included 155 (24.3%) that had quitted smoking successfully and 365 (57.2%) who failed in quitting. The study flowchart is provided in Figure 1.

### Characteristics of participants who were former or current smokers

**Figure 2:**
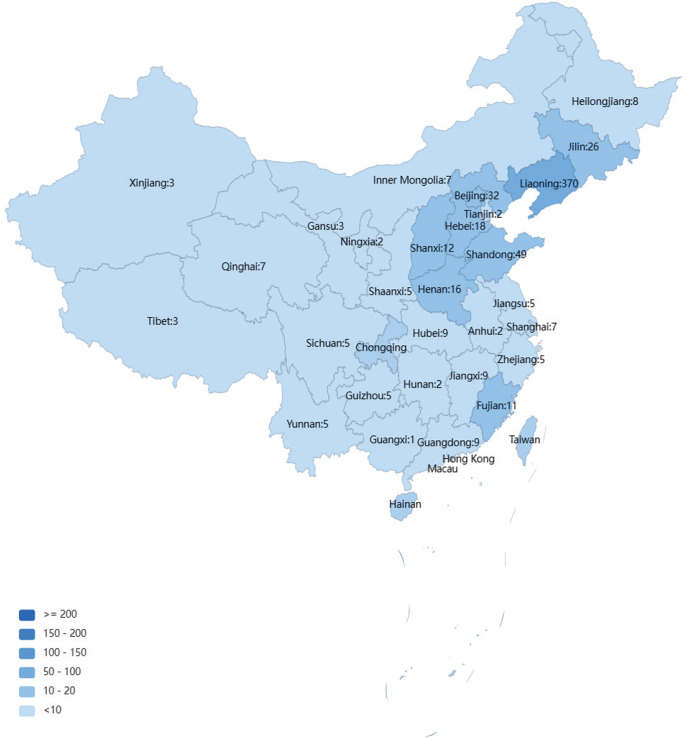
The distribution of provinces of respondents who completed online cross-sectional survey in China from 1 October to 31 November 2022

The 638 participants who were former or current smokers came from 30 provinces (Figure 2) in China, had an average age of 37.3 ± 11.7 years (range: 13–79), and 92.3% were male. The average daily cigarette consumption was 14.8 ± 9.4 (range: 0.5–60), and the smoking history was 15.9 ± 13.7 years (range: 0.5–51).

### Attitudes towards smoking cessation

Regarding the attitude towards smoking cessation, 596 (93.4%) participants had the intention to quit smoking (past: 223, 35.0%; now: 140, 21.9%; future: 71, 11.1%; always: 162, 25.4%). Only 25 (3.9%) of the 638 participants planned never to give up smoking and 17 (2.7%) had not considered this matter.

### Description of those who had at least one previous quit attempt

Of the 520 respondents who had at least one previous quit attempt, 344 (66.9%) had 1–2 quit attempts, 114 (22.5%) had 3–5 quit attempts, and 52 (10.6%) had more than 5 quit attempts. Baseline characteristics of the participants who quitted smoking successfully compared to those who failed in quitting are provided in Table 1.

**Table 1:** Baseline characteristics of the participants who quitted smoking successfully compared to those who were still smoking from 1 October to 31 November 2022 in China

*Characteristics*	*Statistical analysis^a^*				*p*
	*Quit smoking (N=155; 29.81%)*		*Still smoking (N=365; 70.19%)*		
	*Mean*	*SD*	*Mean*	*SD*	
**Age** (years)	39.27	12.83	36.67	11.25	0.030*
**Smoking history** (years)	14.34	11.06	16.55	10.48	0.031*
**Daily cigarette consumption**	13.42	11.44	16.03	8.7	0.012*
**Sex**	n	%	n	%	
Male	137	88.39	340	93.15	0.071
Female	18	11.61	25	6.85	

SD: standard deviation.

* Statistical significance was set at p<0.05.

a Chi-squared test and t-test were used for statistical analysis.

In all, 346 (66.5%) wanted to give up smoking because they had concerns about the general or specific health hazards of smoking, 39 (7.5%) were preparing for pregnancy, 38 (7.3%) were persuaded by relative or friends, 31 (6.0%) had no craving for smoking, 28 (5.4%) had a lack of money, 9 (1.7%) wanted to experience smoking cessation, 6 (1.2%) were required by their work environment. Eight participants gave the reasons to attempt to quit smoking as: bets with friends that they could quit, saving money, academic reasons, decreasing health hazards to others, the smell from smoking, being afraid of a delay to find a spouse, smoking being boring, and that it was an unhygienic habit.

### Description of participants who quitted smoking successfully

The factors that promoted smoking cessation are shown in Table 2. Of the 155 participants who quitted smoking successfully, the smokers themselves (60; 38.7%) and their partners (33; 21.3%) were considered to be of critical importance in the process of quitting smoking. Other family members (25; 15.5%) and child (children) (13; 8.4%) also played important roles in helping smokers to give up smoking. Friends (4; 2.6%); colleagues (3; 1.9%), non-smokers around (3; 1.9%), and healthcare workers (2; 1.3%) also had a positive effect on quitting.

**Table 2:** Factors that promoted smoking cessation in participants who quitted smoking successfully from 1 October to 31 November 2022 in China (N=155)

*Factors*	*n*	*%*	*95% CI*
**Willpower**	86	44.5	0.476–0.634
**Substitutes**	24	15.5	0.097–0.212
Snacking	9	5.8	0.021–0.095
Gum	5	3.2	0.004–0.060
Melon seeds	3	1.9	-0.003–0.041
Tea	3	1.9	-0.003–0.041
Sugar	1	0.6	-0.006–0.019
Water	1	0.6	-0.006–0.019
Alcohol	1	0.6	-0.006–0.019
Coffee	1	0.6	-0.006–0.019
**Diverting attention**	14	9.0	0.045–0.136
Diversion	7	4.5	0.012–0.078
Exercise	4	2.6	0.001–0.051
Watching television	1	0.6	-0.006–0.019
Sleeping	1	0.6	-0.006–0.019
Changing of habits	1	0.6	-0.006–0.019
**Compulsory measures**	7	4.5	0.012–0.078
Supervision	5	3.2	0.004–0.060
Physical discomfort	2	1.3	-0.005–0.031
Pregnancy	1	0.6	-0.006–0.019
**Cutting off sources of tobacco**	6	3.9	0.008–0.069
No cigarettes	5	3.2	0.004–0.060
Throwing away lighters	1	0.6	-0.006–0.019
**Smoking cessation products**	6	3.9	0.008–0.069
Alternatives	3	1.9	-0.003–0.041
E-cigarettes	2	1.3	-0.005–0.031
Smoking cessation spirit	1	0.6	-0.006–0.019
**Psychological interventions**	4	2.6	0.001–0.051
Betting	2	1.3	-0.005–0.031
Psychological suggestion	1	0.6	-0.006–0.019
Self-motivation	1	0.6	-0.006–0.019
**Aversion therapy**	4	2.6	0.001–0.051
Drinking tobacco water	1	0.6	-0.006–0.019
Putting spicy stuff on the cigarette	1	0.6	-0.006–0.019
Reducing the quality of the cigarette	1	0.6	-0.006–0.019
Not taking a shower	1	0.6	-0.006–0.019
**Away from smoking people and environments**	2	1.3	-0.005–0.031
**Reading books related to quitting smoking**	1	0.6	-0.006–0.019

The majority of smokers had different degrees of improvement in their health status after quitting smoking, except for a few people (12; 7.7%) who had mild withdrawal symptoms. It is worth noting that 32 (20.6%) respondents had weight gain after they quit smoking. The positive changes caused by quitting smoking were mainly reflected in the respiratory system (59; 38.1%), physical function and mental status (43; 27.7%), oral environment (7; 4.5%), digestive system (7; 4.5%), and sensory system (4; 2.6%).

### Description of participants who tried to quit but failed

The factors that hindered successful smoking cessation are given in Table 3. When asked which period after quitting smoking they found the most difficult to endure, the overwhelming response from the 365 participants who failed was the first week (133; 36.4%), followed by the first month (31; 8.5%), the first three months (2; 0.5%), and from the third month to a year (3; 0.8%). In addition, the periods of after meals (24; 6.6%) and before bedtime (10; 2.7%) were also the time when respondents were eager to smoke. Relapse was often triggered by specific situations, such as drinking (75; 20.5%), locations where people were smoking (71; 19.5%), parties (67; 18.4%), entertainment (44; 12.1%), places where smoking was not restricted (40; 10.9%), and workplaces (21; 5.8%).

**Table 3:** Factors that hindered smoking cessation in participants who tried to quit but failed from 1 October to 31 November 2022 in China (N=365)

*Factors*	*n*	*%*	*95% CI*
**Lack of willpower**	103	28.2	0.236–0.329
**Tobacco addiction and withdrawal symptoms**	59	16.2	0.124–0.200
Tobacco addiction	36	9.9	0.068–0.129
Psychological dependence	17	4.7	0.025–0.068
Withdrawal symptoms	6	1.6	0.003–0.030
**Environmental impacts**	58	15.9	0.121–0.197
Surrounding smoking population or environment	57	15.6	0.119–0.194
Smoking footage on television	1	0.3	-0.003–0.008
**Bad moods**	36	9.9	0.068–0.129
Bad feelings	24	6.6	0.040–0.091
Boredom and loneliness	12	3.3	0.014–0.051
**Stress**	29	7.9	0.052–0.107
Work stress	20	5.5	0.031–0.078
Life stress	7	1.9	0.005–0.033
Tiredness	2	0.5	-0.002–0.013
**Habits**	26	7.1	0.045–0.098
Habits	24	6.6	0.040–0.091
Staying up late	1	0.3	-0.003–0.008
Playing with the computer	1	0.3	-0.003–0.008
**Socialization**	15	4.1	0.021–0.062
Socialization	7	1.9	0.005–0.033
Socializing	4	1.1	0.000–0.022
Persuading somebody to smoke	4	1.1	0.000–0.022
**Easy availability of tobacco**	10	2.7	0.011–0.044
Having money for cigarettes	7	1.9	0.005–0.033
Tobacco availability anywhere	2	0.5	-0.002–0.013
Constantly launching new tobacco products	1	0.3	-0.003–0.008
**Lack of awareness of tobacco hazards**	6	1.6	0.003–0.030
**No intention to quit smoking**	4	1.1	0.000–0.022
**Lack of compulsory measures**	3	0.8	-0.001–0.018
**Enjoyment**	1	0.3	-0.003–0.008

## DISCUSSION

### Main findings compared with other studies

More than ever, people are aware of tobacco’s harms and consequences^6^. In the present survey, 99.5% of respondents knew about the health hazards of smoking, and 93.4% reported the intention to quit. According to a previous study, 70% of smokers believed that intention was both a necessary and sufficient condition for successful quitting^19^.

Smoking addiction is very difficult resulting in a high rate of relapse^20^, therefore, multiple quit attempts are often required^21^. Evidence has been presented that the number of quit attempts increased the likelihood of relapse^22^. Exploring influencing factors in the preparation phase aims to increase the smoker’s motivation. In the light of our survey, smokers were more likely to formulate an intention to quit smoking action when they perceived its threat to health. Some people stopped smoking during pregnancy, however, they often had high postpartum relapse rates^23^. Advice from relatives or friends, lack of money, experiencing smoking cessation, and requirement by the work environment were the common factors that encouraged smokers to give up smoking. These findings indicate that by strengthening publicity of the health hazards of tobacco, strictly restricting smoking in public places and worksites, increasing monitoring by family, friends and colleagues, and limiting financial expenditure on tobacco, smokers’ quitting motivation can be maintained.

Data from successful quitters showed that willpower was the most important factor for successful quitting, which has been demonstrated in previous studies^24-26^. There is a need to promote smokers’ internal personal driving force and strengthen their willpower from different perspectives and in different ways. Though effective evidence-based interventions are available, the vast majority of smokers tried to quit by themselves based on their own or others’ experiences, and only about 3.9% adopted self-help therapy using smoking cessation products. Moreover, no smoker went to specialist smoking cessation facilities to seek help or treatment. Family members of smokers played prominent roles in facilitating the process of quitting. Studies have shown that advice from health professionals could increase the success rate in quitting smoking^27^. However, through this study, it was found that health professionals did little to help. There are two possible reasons for this phenomenon, one of which is the unavailability and inaccessibility of cessation services^28^, and the other is the lack of awareness among smokers. Many people do not realize that tobacco dependence, a chronic relapsing condition, requires repeated interventions and multiple attempts to get rid of it. What is worse is that a study showed that 35% of the participants believed that use of cessation assistance was a sign of weakness^19^. This study found that quitters experienced varying degrees of health gains after quitting. At the same time, weight gain occurred in 20.6% of quitters and 7.7% had withdrawal symptoms, two important factors for relapse^29,30^.

Previous research has suggested that the main reason for failure to quit smoking was nicotine dependence^29,30^. In the present study, data from 365 people who had failed to quit smoking showed that the biggest barrier to quitting was lack of willpower; however, this can be overcome by the advice of health professionals or encouragement and supervision from family members, friends and colleagues. Tobacco addiction, secondhand smoke exposure, bad moods, stress, habits, and socialization were strongly associated with failure in cessation. In addition, easy availability of tobacco, lack of awareness of tobacco hazards, lack of compulsory measures, lack of motivation, and enjoyment were the barriers to quitting. This study also found that older age appears to be a facilitator of smoking cessation, while smoking history and daily cigarette consumption appear to be barriers to quitting.

The process of smoking cessation consists of three phases: preparation, intervention, and maintenance. Maintenance is necessary for permanent abstinence. Though being engaged in smoking cessation, the relapse of quitters remains extremely high^31^. Evidence regarding influencing factors of relapse after quitting is essential to keep long-term abstinence. This survey showed that the first week after stopping smoking was the most likely time to relapse, which was consistent with previous studies^32,33^. Results indicate that the most frequent smoking relapse situations occurred when smokers were drinking alcohol. Researches have shown that alcohol dependence and tobacco dependence interacted with each other and result in simultaneous withdrawal symptoms^34,35^. Other factors that were closely linked to relapse were secondhand smoke exposure and socialization. The cessation is easily interrupted by other smoking people and environment. In China, people like to give out cigarettes to and receiving cigarettes from others at social occasions as a common social custom. Reversing this social custom is difficult, but essential to tobacco control.

### Potential policy implications

The current survey demonstrated that smokers did not receive help or treatment from healthcare settings in their process of quitting. To resolve this issue, on the one hand, health education must be continuously reinforced to highlight that smoking addiction is a disease and needs to be treated properly. On the other hand, the availability of related healthcare services should be improved, including flexible and diverse smoking cessation establishments and a sufficient number of experienced health professionals. In addition, health insurers can increase cessation rates by covering and promoting evidence-based cessation treatment and removing barriers to accessing treatment. Moreover, there is an urgent need to develop personalized tobacco control program packages targeting different groups of people and cultures. Furthermore, training for family members of smokers should be strengthened to channel smokers’ bad feelings, encourage good habits and reduce failure rates in quitting smoking. It is also very important to promote governmental policies to limit smoking in public places and create smoke-free environments.

### Strengths and limitations

Because of the multiple facets of addiction, factors that influence success in quitting smoking are complex. This study explored the drivers and barriers to successful smoking cessation from three different perspectives: the smokers, the successful quitters, and the failed quitters. These potential factors were explored to the maximum extent by using open-ended questions. Combating smoking addiction requires attention to all these factors. There were some limitations in this research. Firstly, the participants were recruited online, and this may introduce additional selection bias. But the sample characteristics in this research were generally consistent with those described in the 2018 China Adult Tobacco Survey report (the smoking rate among men was 50.5% and 2.1% among women. The average smoker smoked 15.2 cigarettes per day)^36^. This suggests that our sample is representative. Furthermore, the successful cessation was defined as maintaining abstinence for more than six months, but some studies have shown that the relapse rate at one year was higher, so the success rates may be overestimated in this survey. As this was a cross-sectional study, the relationship between different factors to smoking cessation cannot be ascertained.

## CONCLUSIONS

In this study, different potential factors related to smoking cessation have been investigated through open-ended questions. The results suggest that willpower, substitutes, diverting attention, compulsory measures, and cutting off sources of tobacco were the main facilitators promoting quit success. Lack of willpower, tobacco dependence, secondhand smoke exposure, bad moods, stress, habits, socialization, and easy availability of tobacco were the main barriers to successful quitting. Our findings also indicate that an immediate priority may be to upgrade the support and treatment provided by healthcare facilities to smokers who want to quit.

## Data Availability

The data supporting this research are available from the authors on reasonable request.
